# A short length of hospital stay is not associated with risk of readmission among hip fracture patients – a Swedish national register-based cohort study

**DOI:** 10.1186/s12877-023-04464-2

**Published:** 2023-11-15

**Authors:** Stina Ek, Anna C. Meyer, Alexandra Wennberg, Katarina Greve, Margareta Hedström, Karin Modig

**Affiliations:** 1 Unit of Epidemiology, Institute of Environmental Medicine, Karolinska Institutet, Stockholm, Sweden; 2 Department of Clinical Science, Intervention and Technology, Karolinska Institutet, Stockholm, Sweden; 3 Function Perioperative Medicine and Intensive Care (PMI), Karolinska University Hospital, Stockholm, Sweden; 4 Trauma and Reparative Medicine Theme (TRM), Karolinska University Hospital, Stockholm, Sweden

**Keywords:** National registers, Injury, Prognosis, Health care

## Abstract

**Background:**

Hospital length of stay (LoS) after a hip fracture likely mirrors health status; however, a too short hospitalization might increase the risk of readmission. In this national register-based study, we investigated the association between LoS after a hip fracture and the risk of readmissions.

**Methods:**

73,551 patients with a first hip fracture between 2012 and 2019 were followed for 4 months after discharge. LoS was categorized by cubic splines and the association with readmissions was analyzed with Cox regression models.

**Results:**

The mean LoS was 11 ± 6 days and 25% of the study population had at least one readmission. Compared to the mean LoS of 9–12 days, there was a 18% decreased risk of readmission for LoS of 2–4 days (HR 0.82 [95% CI 0.77–0.87]) and 13% decrease for 5–8 days (HR 0.87 [95% CI 0.83–0.91]), when adjusting for sex, age, walking ability, ASA score, CCI, complications during hospitalization and living arrangements. For longer LoS, risk of readmission increased (13–23 days: HR 1.09 [95% CI 1.05–1.13] and 24 + days: HR 1.19 [95% CI 1.11–1.28]). The results were robust across sex, age, and living arrangements. The most common specific reasons for readmission were trauma/injury, cardiovascular and complications, and the proportions did not differ considerably between short and long LoS-categories.

**Conclusions:**

While a long LoS can be explained by the care need of the patient, a short LoS - compared to the average stay - does not increase the risk of readmission regardless of health status and hospital complications in a Swedish setting.

**Supplementary Information:**

The online version contains supplementary material available at 10.1186/s12877-023-04464-2.

## Background

Hip fracture patients have high excess mortality, with twice as high mortality risk compared to their peers [1]. Various factors are associated with a poor prognosis after a hip fracture, although some are still debated. The length of hospital stay (LoS) in conjunction with the fracture and the association with 30-days mortality have shown diverse results, with studies showing increased risk for both short and long LoS. One study from the US showed that a longer LoS is associated to higher mortality [2], while both a South Korean and a Swedish study showed that a very short LoS increased the risk of death [3, 4]. However, a comparison of these studies must be done with caution due to differences in healthcare systems between regions and countries. The main differences are whether the health care is paid for by the state or the individual, and whether the rehabilitation takes place at the hospital or in another setting.

In a previous study, we showed that a longer LoS increased the risk of mortality over a longer follow-up of 4 months, while a short LoS did not. However, patients living in care homes were often discharged early (from the hospital back to their care home) while also being at higher risk of short-term death than patients living independently [5]. Mortality is, however, the ultimate outcome and it is often preceded by complications and re-admission to hospital. It is therefore of clinical importance to analyze the risk of hospital readmissions in relation to LoS as well. Several studies have shown that a longer LoS is associated with an increased risk of readmissions [6–9]; although most studies display weak associations [10], one study has shown that a longer LoS decreases the risk of readmissions [11]. While a longer hospitalization likely reflects the patient’s poorer health status, and the increased risk of mortality and readmissions, the reasons behind a short LoS are less straight forward. A relatively healthy individual might be discharged early because they are already stable and mobilized a few days after hip fracture surgery, while a frailer individual might not be mobilized immediately and is at risk of acute adverse outcomes such as pneumonia. One might also hypothesize that individuals living in care homes are being discharged early because they can receive both care and rehabilitation on site. However, there may be situations where a short LoS is not representative of the patient’s health status or access to care after discharge. A lack of hospital beds or economic incentives might lead to early discharge even for vulnerable patients who will consequently be at higher risk of a hospital readmission.

It is important to determine whether a shorter LoS than the mean (which can be considered the norm) introduces a higher risk of complications and consequently readmissions, and if so, among which patients. We have seen that the association between short LoS and mortality differs between patient groups based on sociodemographic- and health-related profiles [5], however, there is a lack of studies investigating such a difference in the association between short LoS and readmissions.

We therefore performed a population-based cohort study among hip fracture patients investigating the association between LoS and readmissions during a 4-month period, considering the role of sociodemographic- and health-related factors of the individuals.

## Methods

### Data

All individuals in Sweden above the age of 65 admitted to the hospital with an incident hip fracture between the years 2012–2019 were identified in the Swedish National Patient Register (NPR), using ICD-10 codes S720-S722. Information on the outcome of death was extracted from the Cause of Death Register and information on sociodemographic factors and health status was extracted from RIKSHÖFT, the Swedish National Registry for Hip Fractures (SHR). The different sources were linked to each other with the Swedish Personal Number (PIN) assigned to all residents in Sweden. The NPR and the Cause of Death Register are administrative registers with close to full national coverage [12], while the SHR is a clinical register with a coverage of 80–90% of all hip fractures in Sweden during the study period [13].

In total, 91,383 hip fractures were identified in both NPR and SHR. We excluded patients that did not receive surgery or that had a pathologic fracture (n = 195 and n = 1,015, respectively), due to expected differences in health care utilization, as well as individuals who had excessively short or long hospitalizations (LoS < 2 n = 575, LoS > 30 n = 3,446). We also excluded those who died during the initial hospital stay (n = 3,784) or were transferred to another clinic or hospital (n = 5,436), because the time to event started at discharge from the hospital. Last, individuals with missing values in any of the included variables were excluded (n = 3,411). The final analytical sample consisted of 73,551 individuals aged 65 years or older who had endured their first hip fracture anytime during the period 2012–2019.

The exposure of LoS was based on data from the NPR and was calculated in days, spanning from the day that the patient was admitted to a hospital due to a hip fracture until the day the patient was discharged.

The outcome was time until being readmitted to a hospital during the first 4 months after being discharged from the index hospitalization. This data was gathered from the NPR. Reasons for readmission were based on ICD10-codes and were categorized into: infections, tumors, endocrine disorders, psychological disorders (including dementia), neurological diseases, cardiovascular events, respiratory conditions, injuries and trauma, check-up/follow-up visit, complications not otherwise specified (NOS), and other/NOS.

Covariates were age, sex, as well as the Charlson Comorbidity Index (CCI) and complications during hospital stay which were gathered from the NPR. CCI was calculated using data from 5 years prior to the hip fracture according to Ludvigsson et al. [14], and a CCI score more than 4 was merged into one category. Complications at the hospital stay could had emerged during the stay or been present when the patient arrived at the hospital and included pneumonia, urinary tract infection, thrombosis, delirium, decubitus, and any ICD codes including complications from surgery (such as infections). American Society of Anaesthesiologists physical status classification (ASA score) [15], walking ability, and living arrangements before admission were retrieved from the SHR. In this study, ASA scores of 4 and 5 were merged into the same category. Walking ability was self-reported and categorized into five categories: independent, assisted outside, independent inside, assisted inside, not able to walk. The individual’s living arrangement before hip fracture admission was categorized into three categories: independent living/care home or other type of service facility/other health care facility (other hospital or another ward). Type of fracture (categorized as cervical or intertrochanteric/subtrochanteric) and type of surgery (screws, nails, or side plate/intramedullary nail/arthroplasty - hemi or total) was also retrieved from the SHR. Dementia was either recorded in the SHR at the index hospitalization or a dementia diagnosis in the NPR 5 years prior to the index hospitalization.

### Statistical analysis

Descriptive information about the study population was stratified by LoS categories and presented in percentages or means. We used restricted cubic splines to divide the exposure of LoS in days into a variable containing five categories: 2–4 days, 5–8 days, 9–12 days, 13–23 days, and 24–30 days). Absolute risks of readmission within 4 months for the different categories of LoS were calculated for all and stratified by sex, age groups, and living arrangement. The risk of readmission over time was analyzed with Cox proportional hazards models, with the categories of LoS as exposure, using the category including the mean LoS for this population (9–12 days) as a reference. Model 1 was adjusted for sex and age, and Model 2 was adjusted for sex, age, ASA score, CCI, complication at the hospital, walking ability, and living arrangement before admission. The models were also stratified by sex, age groups (young = 60–79 years, old = 80 + years), and living arrangements before index admission, since we have seen differences between these subgroups in previous research [5]. In the stratified analysis for living arrangements the ‘care home’ category and ‘health care facility’ categories were merged into one group.

### Sensitivity analyses

We hypothesized that the mechanisms behind readmissions would be similar to those for mortality and therefore performed a competing event analysis with readmissions as event of interest and death as competing event. This would be particularly pertinent among care home residents, who more often die in the care home without being admitted to a hospital in the terminal phase, and therefore have the competing event “instead of” the main event. The competing event analysis is a specific type of survival analysis that gives a marginal probability of the outcome in presence of another outcome that is highly probable to happen during the study period, a competing event. The competing event analysis is less likely to overestimate the risk of the outcome [16]. In addition to the competing event analysis, we performed survival analysis stratified by type of fracture, surgery method, and ASA score. Severity of fracture and following surgery method might have an impact on LoS in the way that more severe fractures have a longer LoS and an increased risk of readmissions, and thus confound the association between LoS and prognosis. Last, an analysis among individuals with diagnosed dementia was performed, since cognitive status might affect the health care utilization.

Statistical analyses were performed with Stata 16 (StataCorp. 2019, College Station, TX).

### Patient and public involvement

Since this study is based on anonymized register data collected over several years, no involvement of patients was possible at the time of the study.

## Results

**Fig. 1:**
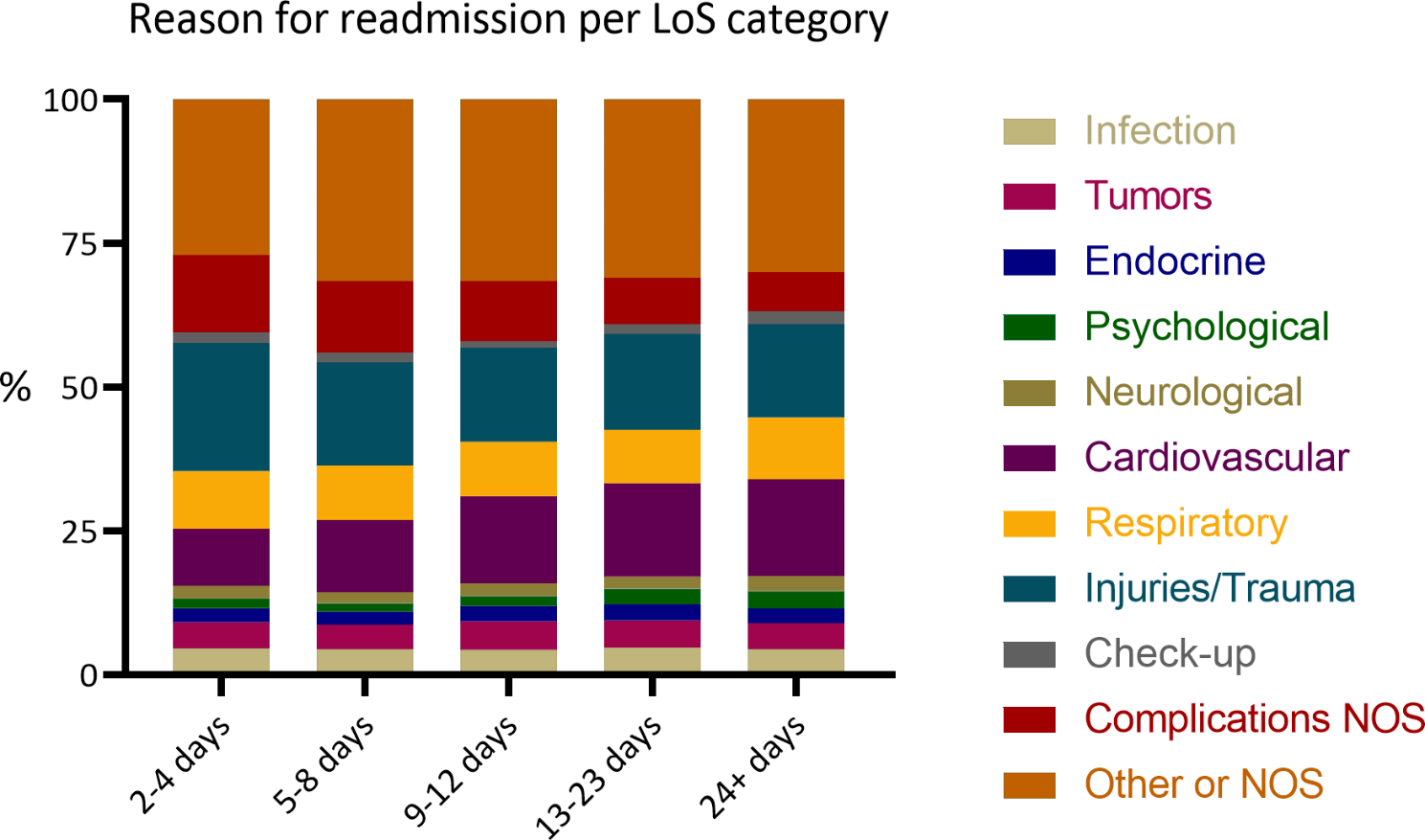
Reason for the first readmission among participants with any readmission within 4 months from index discharge, n = 18,638. NOS: unspecified

The mean age of the hip fracture patients was 83 ± 7 years and almost 70% were women. Most individuals lived independently at the time of the fracture, walked independently, and had an ASA score of 2 or 3, as seen in Table 1. The mean LoS was 10.6 ± 6 days and 25% of the study population had at least one readmission to a hospital during the 4 months of follow-up. During the study period, the mean LoS decreased from 11.4 days in 2012 to 9 days in 2019, see Supplementary Fig. 1. Patients with a LoS below the mean were slightly younger and had lower ASA score, but at the same time a lower walking ability, more often resided in care homes, and had a higher death rate. Patients in the reference and longer LoS categories were similar in terms of age and health indicators. The most common diagnoses for readmission were injuries/trauma or cardiovascular disease, although the most readmissions were categorized as “other or NOS” which means that we do not know their reason for readmission. The distribution of main diagnosis for readmission for each LoS category is presented in Fig. 1. Being readmitted to a hospital was more common among those with longer LoS – 34% among the group with 24 + days compared to 19% in the group with 2–4 days. The association between LoS and time to readmission had a dose-response pattern so that each category of longer LoS had an increase in the HR for readmission compared to a shorter LoS. Compared to the reference group (LoS 9–12 days), a shorter LoS was associated with a lower relative risk of readmission (2–4 days HR 0.76 [95% CI 0.72–0.80] and 5–8 days HR 0.84 [95% CI 0.81–0.87]), while longer LoS was associated with a higher risk of readmission within 4 months (13–23 days HR 1.16 [95% CI 1.12–1.21] and 24 + days HR 1.35 [95% CI 1.26–1.45]) in Model 1. Additionally adjusting for walking ability, ASA score, CCI, complications during hospitalization, and living arrangements in Model 2 did not substantially alter the results: 2–4 days HR 0.82 [95% CI 0.77–0.87], 5–8 days HR 0.87 [95% CI 0.83–0.91], 13–23 days HR 1.09 [95% CI 1.05–1.13], 24 + days HR 1.19 [95% CI 1.11–1.28], compared to 9–12 days.

**Table 1:** Baseline characteristics of the whole study population and divided by LoS categories, presented as number (percentage), or mean (standard deviation)

	All (n = 73,551)	LoS 2–4 days (n = 8,982)	LoS 5–8 days (n = 21,723)	LoS 9–12 days (n = 19,913)	LoS 13–23 days (n = 20,123)	LoS 24 + days (n = 2,810)
**Age, mean (SD)**	83.2 (7.8)	82.1 (8.6)	82.7 (8.0)	83.2 (7.5)	83.9 (7.4)	83.9 (7.2)
**Women, n (%)**	50,928 (69.2)	6,133 (68.3)	15,331 (70.6)	13,902 (69.8)	13,747 (68.3)	1,815 (64.6)
**ASA score, n (%)**						
1	3,286 (4.5)	631 (7.0)	1,193 (5.5)	847 (4.3)	559 (2.8)	56 (2.0)
2	27,195 (37.0)	3,324 (37.0)	8,323 (38.3)	7,849 (39.4)	6,847 (34.0)	852 (30.3)
3	38,334 (52.1)	4,410 (49.1)	10,898 (50.2)	10,091 (50.7)	11,304 (56.2)	1,631 (58.0)
4–5	4,736 (6.4)	617 (6.9)	1,309 (6.0)	1,126 (5.7)	1,413 (7.0)	271 (9.6)
**CCI, n (%)**						
**0**	31,974 (43.5)	3,868 (43.1)	9,728 (44.8)	9,083 (45.6)	8,232 (40.9)	1,063 (37.8)
**1**	15,992 (21.7)	2,377 (26.5)	4,866 (22.4)	3,987 (20.0)	4,193 (20.8)	569 (20.3)
**2**	10,632 (14.5)	1,218 (13.6)	3,124 (14.4)	2,885 (14.5)	2,991 (14.9)	414 (14.7)
**3**	5,889 (8.0)	678 (7.6)	1,655 (7.6)	1,522 (7.6)	1,736 (8.6)	298 (10.6)
**4+**	9,064 (12.3)	841 (9.4)	2,350 (10.8)	2,436 (12.2)	2,971 (14.8)	466 (16.6)
**Complication at the hospital**	10,722 (14.6)	385 (4.3)	1,716 (7.9)	2,512 (12.6)	5,078 (25.2)	1,031 (36.7)
**Walking ability before admission, n (%)**						
Independent	45,432 (61.8)	3,940 (43.9)	12,203 (56.2)	13,692 (68.8)	13,719 (68.2)	1,878 (66.8)
Assisted outside	6,415 (8.7)	1,012 (11.3)	1,904 (8.8)	1,496 (7.5)	1,755 (8.7)	248 (8.8)
Independent inside	15,105 (20.5)	2,508 (27.9)	5,063 (23.3)	3,463 (17.4)	3,556 (17.7)	515 (18.3)
Assisted inside	4,692 (6.4)	1,038 (11.6)	1,864 (8.6)	897 (4.5)	771 (3.8)	122 (4.3)
Not able to walk	1,907 (2.6)	484 (5.4)	689 (3.2)	365 (1.8)	322 (1.6)	47 (1.7)
**Coming from (at admission to hospital), n (%)**						
Independent living	52,856 (71.9)	3,340 (37.2)	12,809 (59.0)	16,561 (83.2)	17,665 (87.8)	2,481 (88.3)
Care home or similar	17,815 (24.2)	5,330 (59.3)	8,193 (37.7)	2,623 (13.2)	1,532 (7.6)	17,815 (24.2)
Other health care facility	2,880 (3.9)	312 (3.5)	721 (3.3)	729 (3.7)	926 (4.6)	2,880 (3.9)
**4-month mortality, n (%)**	9,200 (12.5)	1,599 (17.8)	3,019 (13.9)	2,013 (10.1)	2,184 (10.9)	385 (13.7)
**30-days readmission, n (%)**	9,389 (12.8)	960 (10.7)	2,490 (11.5)	2,549 (11.5)	2,893 (14.4)	497 (17.7)
**4-month readmission, n (%)**	18,638 (25.3)	1,729 (19.3)	4,683 (21.6)	5,197 (26.1)	6,073 (30.2)	956 (34.0)
Cause of readmission, n (%) *						
Infection	834 (4.5)	79 (4.6)	207 (4.4)	223 (4.3)	283 (4.7)	42 (4.4)
Tumours	865 (4.7)	79 (4.6)	197 (4.2)	258 (5.0)	288 (4.8)	43 (4.5)
Endocrine	478 (2.6)	41 (2.4)	109 (2.3)	138 (2.7)	166 (2.7)	24 (2.5)
Psychological incl dementia	374 (2.0)	29 (1.7)	64 (1.4)	86 (1.7)	166 (2.7)	29 (3.0)
Neurological	397 (2.1)	38 (2.2)	90 (1.9)	115 (2.2)	129 (2.1)	25 (2.6)
Cardiovascular	2,681 (14.4)	171 (9.9)	585 (12.5)	784 (15.2)	981 (16.2)	160 (16.8)
Respiratory	1,768 (9.5)	174 (10.1)	442 (9.5)	490 (9.5)	559 (9.2)	103 (10.8)
Injuries and trauma	3,235 (17.4)	386 (22.4)	838 (18.0)	848 (16.4)	1,008 (16.7)	155 (16.3)
Check-up/follow-up visit	294 (1.6)	30 (1.7)	80 (1.7)	57 (1.1)	106 (1.8)	21 (2.2)
Complications NOS	1,906 (10.3)	233 (13.5)	582 (12.5)	543 (10.5)	484 (8.0)	64 (6.7)
Other or NOS	5,739 (30.9)	467 (27.0)	1,473 (31.6)	1,632 (31.5)	1,880 (31.1)	287 (30.1)

ASA = American Society of Anaesthesiologists physical status classification; CCI = Charlson Comorbidity Index; UNS = unspecified; *subsample of readmissions within 4 months and non-missing ICD code, n = 12,092

**Fig. 2:**
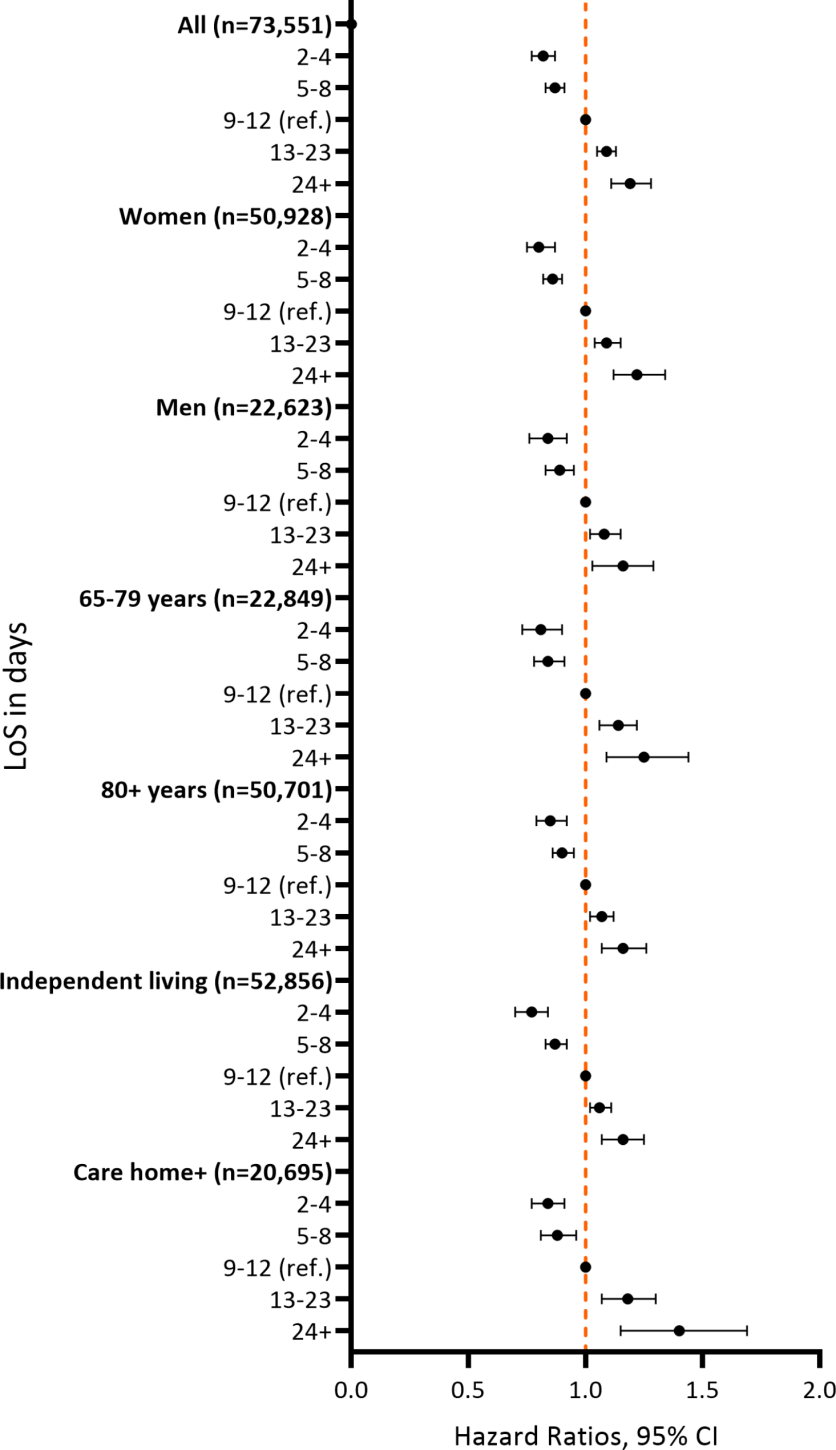
Association between categories of LoS and 4-month readmissions, analyzed with Cox proportional hazards (HR, 95% significance level), adjusted for age, sex, walking ability before the fracture, ASA score, CCI, complications during hospitalization, and living arrangements before the fracture

When analyzing the association between LoS and risk of readmission for different subgroups specifically, similar associations were found for women and men, different age groups and for different living arrangements (Table 2; Fig. 2).

**Table 2:** Absolute risk and hazard ratios (95% CI) between categories of LoS and 4-month readmissions, for the full sample and stratified by; sex, age groups, and living arrangements prior to admission

LoS, in days	Absolute risk			Hazard ratio (95% CI)	
	n	cases	%	Model 1	Model 2
**All**	**73,551**	**18,638**	**25.3**		
2–4	8,982	1,729	19.3	**0.76 (0.72–0.80)**	**0.82 (0.77–0.87)**
5–8	21,723	4,683	21.6	**0.84 (0.81–0.87)**	**0.87 (0.83–0.91)**
9–12	19,913	5,197	26.1	Ref	Ref
13–23	20,123	6,073	30.2	**1.16 (1.12–1.21)**	**1.09 (1.05–1.13)**
24+	2,810	956	34.0	**1.35 (1.26–1.45)**	**1.19 (1.11–1.28)**
**Women**	**50,928**	**11,791**	**23.2**		
2–4	6,133	1,057	17.2	**0.74 (0.69–0.79)**	**0.80 (0.75–0.87)**
5–8	15,331	2,982	19.5	**0.82 (0.78–0.86)**	**0.86 (0.82–0.90)**
9–12	13,902	3,320	23.9	Ref	Ref
13–23	13,747	3,853	23.9	**1.19 (1.13–1.25)**	**1.09 (1.04–1.15)**
24+	1,815	579	31.9	**1.40 (1.28–1.53)**	**1.22 (1.12–1.34)**
**Men**	**22,623**	**6,847**	**30.3**		
2–4	2,849	672	23.6	**0.80 (0.73–0.87)**	**0.84 (0.76–0.92)**
5–8	6,392	1,701	26.6	**0.87 (0.81–0.87)**	**0.89 (0.83–0.95)**
9–12	6,011	1,877	31.2	Ref	Ref
13–23	6,376	2,220	34.8	**1.13 (1.06–1.20)**	**1.08 (1.02–1.15)**
24+	995	377	37.9	**1.29 (1.15–1.44)**	**1.16 (1.03–1.29)**
**Age 65–79**	**22,849**	**5,170**	**22.6**		
2–4	3,437	590	17.2	**0.71 (0.65–0.78)**	**0.81 (0.73–0.90)**
5–8	7,411	1,389	18.7	**0.79 (0.73–0.85)**	**0.84 (0.78–0.91)**
9–12	5,978	1,404	23.5	Ref	Ref
13–23	5,306	1,539	29.0	**1.26 (1.17–1.36)**	**1.14 (1.06–1.22)**
24+	717	248	34.6	**1.55 (1.35–1.77)**	**1.25 (1.09–1.44)**
**Age 80+**	**50,701**	**13,468**	**26.6**		
2–4	5,545	1,139	20.5	**0.80 (0.74–0.85)**	**0.85 (0.79–0.92)**
5–8	14,311	3,294	23.0	**0.86 (0.83–0.91)**	**0.90 (0.86–0.95)**
9–12	13,935	3,793	27.2	Ref	Ref
13–23	14,817	4,534	30.6	**1.14 (1.09–1.19)**	**1.07 (1.02–1.12)**
24+	2,093	708	33.8	**1.30 (1.20–1.40)**	**1.16 (1.07–1.26)**
**Independent living**	**52,856**	**13,636**	**25.8**		
2–4	3,340	533	16.0	**0.64 (0.58–0.70)**	**0.77 (0.70–0.84)**
5–8	12,809	2,675	20.9	**0.81 (0.78–0.86)**	**0.87 (0.83–0.92)**
9–12	16,561	4,314	26.1	Ref	Ref
13–23	17,665	5,282	29.9	**1.15 (1.11–1.20)**	**1.06 (1.02–1.11)**
24+	2,481	832	33.5	**1.33 (1.24–1.43)**	**1.16 (1.07–1.25)**
**Care home**	**20,695**	**5,002**	**24.2**		
2–4	5,642	1,196	21.2	**0.81 (0.74–0.88)**	**0.84 (0.77–0.91)**
5–8	8,914	2,008	22.5	**0.86 (0.80–0.94)**	**0.88 (0.81–0.96)**
9–12	3,352	883	26.3	Ref	Ref
13–23	2,458	791	32.2	**1.22 (1.11–1.34)**	**1.18 (1.07–1.30)**
24+	329	124	37.7	**1.48 (1.22–1.78)**	**1.40 (1.15–1.69)**

Model 1: adjusted for age and sex, Model 2: additionally adjusted for walking ability before the fracture, ASA score, CCI, complications during hospitalization, and living arrangements

### Sensitivity analysis

The results from the competing event analysis were similar to the Cox model estimates and the fully adjusted models for the competing event analysis compared to the Cox are shown in Supplementary Table 1. The HRs from the sensitivity analysis stratified for fracture- and surgery type and ASA score showed similar estimates as the main analyses. There was a constant protective effect of a short LoS compared to longer ones. The effect size was the same over fracture types, whereas the effect size was slightly more pronounced for surgery with screws, nails, or plate compared to intramedullary nail or arthroplasty. Individuals with dementia had a similar association as the main analysis, albeit only with a significantly increased risk for the 13–23 days category in the fully adjusted model. (Supplementary Table 2).

## Discussion

In this nationwide Swedish cohort study of hip fracture patients, we found a dose-response relationship between LoS and the risk of readmission to hospital within 4 months from discharge. While it is expected that a long LoS is related to adverse health outcomes, this study adds to the literature by showing that a short LoS is not associated with an increased risk of readmission in relation to an average LoS. The associations were robust for both women and men, younger and older patients, and for different living arrangements prior to the hospitalization. This deviates from studies investigating LoS and mortality, where the associations were higher among both patients with a very short LoS as well as among those with a long LoS [5]. The results were further strengthened by the results from the competing event analysis that did not show any major differences to the main Cox analyses. This suggests that competing risk of death among those with a short hospitalization time did not explain that this group did not have an increased risk of readmission, possibly because in many cases readmission will happen prior to the competing event of death [17].

Our results reinforce the body of evidence showing that staying at the hospital for a long period increases the risk of rehospitalizations [2, 6–9], likely because those who stay long do so because they need more care, have poorer health and are at a higher risk of hospitalizations in general. Therefore, we would like to emphasize the findings that a short LoS (compared to the mean LoS) did not increase the risk of readmission, even in the most vulnerable groups, such as the oldest old, those living in care homes and those with more severe fractures and complicated surgery methods.

Even if we adjust and stratify for several confounding factors related to initial health status such as age, sex, ASA grade, comorbidity, walking ability, and living arrangements, we cannot rule out residual confounding from acute events. That is why we also adjusted for complications that appeared during the hospitalization, in an effort to capture also acute events that might affect both LoS and the risk of readmission. Another important factor for both prognosis after hip fracture and LoS is frailty [18]. Although not adjusted for in this study, we believe that other factors adjusted for in the analyses can act as proxies for frailty, such as age, comorbidity, complications during the hospitalization and living arrangements.

A possible explanation to why a short hospital stay could be favorable might be that individuals are more likely to avoid nosocomial infections, a risk that increases with longer hospital stay and leads to adverse outcomes, even in the long-term [19]. Coming back to one’s familiar environment as soon as possible might also decrease the risk of acute confusion or delirium, a condition that has been shown to increase the risk of readmission [20].

The diagnoses for readmissions did not differ much between the short and long LoS groups, and the main part consisted of unspecified or very broad diagnoses, although there were some small differences between the LoS categories. ‘Injuries/trauma’ and ‘complications’ were more common among those with short LoS, while ‘cardiovascular’ conditions were more common among individuals with long LoS. This indicates that the individuals with a longer LoS were frail and/or had other comorbidities that could require hospitalizations as well, while the readmissions for the shorter LoS groups were driven by more acute and unforeseen events. The higher frequency of ‘injuries/trauma’ and ‘complications’ in the short LoS categories can be interpreted as a sign that they were discharged too early. It is likely that a small group of those with a short LoS would have benefited from staying longer in the hospital, thus avoiding injurious falls or similar events shortly after discharge [21]. However, when interpreting these differences, it is also important to remember that the absolute risk for readmissions was greater in the reference LoS group – 19% readmitted in the shortest LoS category compared to 26% in the mean LoS category.

The strengths of this study include large, nationwide, and thus representative data from a combination of administrative and clinical registers. We were able to investigate the association in-depth among different strata of the Swedish hip fracture population, to test the robustness of our results. However, retrieving data from registers did not allow us to adjust for possible residual confounding, such as lifestyle factors. One key factor for LoS ought to be the severity of the fracture. Although we did not have direct information about severity of fracture, we did have information about fracture type and surgical method, two variables that largely capture fracture severity. However, the sensitivity analyses in strata of different types of fractures- and surgical types showed similar results as the main analysis. Therefore, we believe that the possible residual confounding from fracture severity does not seem to be of major importance. Other important factors are surgical delay and how fast the patient was mobilized, and an early initiating rehabilitation, a key factor to good prognosis after a hip fracture [22]. However, in Sweden, the waiting time for surgery is short and there are strict guidelines for a fast mobilization after surgery [23, 24]. These circumstances, and other factors such as where the rehabilitation takes place and what the possibilities for further care are after discharge, differ substantially between countries and even regions. Therefore, when interpreting the results from this study and comparing them to previous research, one must consider that all regions and countries have different health care systems and guidelines, thus results from one system are not directly applicable to another. The LoS in Sweden is relatively low, partly because the rehabilitation happens in specialized short-term care homes or in the individuals’ own home utilizing Sweden’s home care. Nevertheless, the association between LoS and readmission to hospital can be similar, despite differences in mean LoS, if the mechanisms are the same.

In summary, with this study among hip fracture patients we show that the association between LoS and readmission to a hospital within 4 months shows a dose-response relationship with a lower risk for shorter LoS. These findings were robust across different strata of sex, age, and living arrangement. This suggests that, while a long LoS is explained by the patient’s higher care needs, a shorter LoS than the average does not seem to increase the risk for readmission to hospital, regardless of the patients’ health status and complications during the hospitalization.

### Electronic supplementary material

Below is the link to the electronic supplementary material.

Supplementary Material 1

## Data Availability

The datasets generated and analyzed during the current study are not publicly available due to national GDPR regulations, please contact the corresponding author with requests.
