# Supplementary material for: A short length of hospital stay is not associated with risk of readmission among hip fracture patients – a Swedish national register-based cohort study
Source: BMC Geriatr. 2023 Nov 15;23:744. doi: 10.1186/s12877-023-04464-2 (PMC10648637; doi:10.1186/s12877-023-04464-2)
Supplement: Supplementary file 1 — Supplementary Material 1 [file 12877_2023_4464_MOESM1_ESM.docx]

Supplementary Material


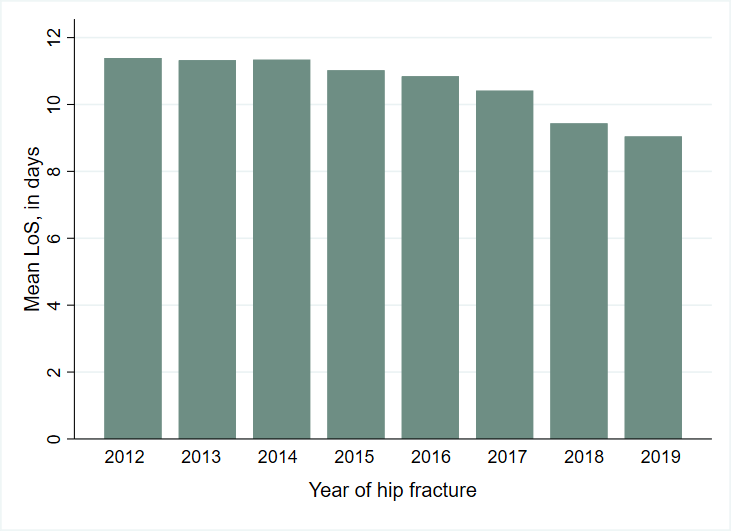


Supplementary Figure 1. Mean LoS per calendar year of hip fracture during the study period of 2012-2019.

Supplementary Table 1. Sub-hazard ratios (95% CI) and hazard ratios between categories of LoS and 4-month readmissions, adjusted for age, sex, walking ability before the fracture, ASA score, CCI, complications during index hospitalization, and living arrangements before the fracture.

| **LoS, in days** | **Competing event analysis** | | | **Cox** | |
| --- | --- | --- | --- | --- | --- |
|  |  | SHR |  |  | HR |
| **All** | *N=73,551, cases: 14,827, competing events: 9,187* | | | |  |
| 2-4 |  | **0.79 (0.74-0.84)** |  |  | **0.82 (0.77-0.87)** |
| 5-8 |  | **0.87 (0.83-0.91)** |  |  | **0.87 (0.83-0.91)** |
| 9-12 |  | Ref |  |  | Ref |
| 13-23 |  | **1.09 (1.04-1.13)** |  |  | **1.09 (1.05-1.13)** |
| 24+ |  | **1.14 (1.05-1.23)** |  |  | **1.19 (1.11-1.28)** |
| **Women** | *N=50,928, cases: 9,622, competing events: 5,625* | | | |  |
| 2-4 |  | **0.79 (0.72-0.86)** |  |  | **0.80 (0.75-0.87)** |
| 5-8 |  | **0.85 (0.80-0.90)** |  |  | **0.86 (0.82-0.90)** |
| 9-12 |  | Ref |  |  | Ref |
| 13-23 |  | **1.10 (1.04-1.16)** |  |  | **1.09 (1.04-1.15)** |
| 24+ |  | **1.18 (1.07-1-30)** |  |  | **1.22 (1.12-1.34)** |
| **Men** | *N=22,623, cases: 5,205, competing events: 3,562* | | | |  |
| 2-4 |  | **0.79 (0.70-0.88)** |  |  | **0.84 (0.76-0.92)** |
| 5-8 |  | **0.90 (0.83-0.97)** |  |  | **0.89 (0.83-0.95)** |
| 9-12 |  | Ref |  |  | Ref |
| 13-23 |  | 1.06 (0.99-1.14) |  |  | **1.08 (1.02-1.15)** |
| 24+ |  | 1.08 (0.95-1.23) |  |  | **1.16 (1.03-1.29)** |
| **Age 65-79** | *N=22,849, cases: 4,466, competing events: 1,246* | | | |  |
| 2-4 |  | **0.77 (0.69-0.86)** |  |  | **0.81 (0.73-0.90)** |
| 5-8 |  | **0.85 (0.78-0.92)** |  |  | **0.84 (0.78-0.91)** |
| 9-12 |  | Ref |  |  | Ref |
| 13-23 |  | **1.14 (1.05-1.23)** |  |  | **1.14 (1.06-1.22)** |
| 24+ |  | **1.23 (1.06-1.43)** |  |  | **1.25 (1.09-1.44)** |
| **Age 80+** | *N=50,701, cases: 10,361, competing events: 7,940* | | | |  |
| 2-4 |  | **0.86 (0.78-0.93)** |  |  | **0.85 (0.79-0.92)** |
| 5-8 |  | **0.91 (0.86-0.96)** |  |  | **0.90 (0.86-0.95)** |
| 9-12 |  | Ref |  |  | Ref |
| 13-23 |  | **1.06 (1.01-1.11)** |  |  | **1.07 (1.02-1.12)** |
| 24+ |  | 1.09 (0.99-1.20) |  |  | **1.16 (1.07-1.26)** |
| **Independent living** | *N=52,856, cases: 11,438, competing events: 3,787* | | | |  |
| 2-4 |  | **0.78 (0.71-0.86)** |  |  | **0.77 (0.70-0.84)** |
| 5-8 |  | **0.88 (0.83-0.92)** |  |  | **0.87 (0.83-0.92)** |
| 9-12 |  | Ref |  |  | Ref |
| 13-23 |  | **1.06 (1.01-1.11)** |  |  | **1.06 (1.02-1.11)** |
| 24+ |  | **1.09 (1.01-1.19)** |  |  | **1.16 (1.07-1.25)** |
| **Care home** | *N=20,695, cases: 3,389, competing events: 5,400* | | | |  |
| 2-4 |  | **0.76 (0.68-0.85)** |  |  | **0.84 (0.77-0.91)** |
| 5-8 |  | **0.85 (0.77-0.93)** |  |  | **0.88 (0.81-0.96)** |
| 9-12 |  | Ref |  |  | Ref |
| 13-23 |  | **1.21 (1.08-1.36)** |  |  | **1.18 (1.07-1.30)** |
| 24+ |  | **1.42 (1.14-1.78)** |  |  | **1.40 (1.15-1.69)** |

Supplementary Table 2. Hazard ratios (95% CI) between categories of LoS and 4-month readmissions, stratified by; type of fracture, type of surgery and ASA score.

| **LoS, in days** | **Hazard Ratios, 95% CI** | |
| --- | --- | --- |
|  | Model 1 | Model 2 |
| **Individuals with dementia, n=15,492** | | |
| 2-4 | **0.83 (0.75-0.92)** | 0.94 (0.84-1.05) |
| 5-8 | **0.89 (0.81-0.97)** | 0.96 (0.87-1.05) |
| 9-12 | Ref | Ref |
| 13-23 | **1.20 (1.08-1.34)** | **1.14 (1.02-1.27)** |
| 24+ | **1.30 (1.06-1.60)** | 1.18 (0.96-1.46) |
| **Type of fracture** | | |
| **Cervical** |  |  |
| 2-4 | **0.76 (0.71-0.81)** | **0.82 (0.76-0.88)** |
| 5-8 | **0.81 (0.77-0.86)** | **0.85 (0.80-0.90)** |
| 9-12 | Ref | Ref |
| 13-23 | **1.19 (1.13-1.25)** | **1.11 (1.06-1.17)** |
| 24+ | **1.37 (1.24-1.51)** | **1.19 (1.07-1.32)** |
| **Intertrochanteric/Subtrochanteric** | |  |
| 2-4 | **0.78 (0.72-0.85)** | **0.83 (0.76-0.92)** |
| 5-8 | **0.87 (0.82-0.93)** | **0.90 (0.85-0.96)** |
| 9-12 | Ref | Ref |
| 13-23 | **1.14 (1.08-1.20)** | **1.06 (1.01-1.12)** |
| 24+ | **1.34 (1.22-1.47)** | **1.19 (1.08-1.31)** |
| **Type of surgery** | | |
| **Screws, nails, or plate** | |  |
| 2-4 | **0.66 (0.61-0.72)** | **0.73 (0.67-0.80)** |
| 5-8 | **0.80 (0.75-0.85)** | **0.83 (0.78-0.89)** |
| 9-12 | Ref | Ref |
| 13-23 | **1.14 (1.07-1.21)** | 1.06 (0.99-1.13) |
| 24+ | **1.28 (1.13-1.45)** | **1.15 (1.01-1.30)** |
| **Intramedullary nail** |  |  |
| 2-4 | **0.80 (0.71-0.90)** | 0.85 (0.75-0.97) |
| 5-8 | **0.85 (0.78-0.92)** | **0.87 (0.80-0.95)** |
| 9-12 | Ref | Ref |
| 13-23 | **1.14 (1.06-1.23)** | **1.07 (0.99-1.15)** |
| 24+ | **1.39 (1.24-1.56)** | **1.21 (1.08-1.36)** |
| **Arthroplasty** |  |  |
| 2-4 | **0.87 (0.79-0.96)** | 0.92 (0.83-1.02) |
| 5-8 | **0.87 (0.82-0.93)** | **0.91 (0.85-0.97)** |
| 9-12 | Ref | Ref |
| 13-23 | **1.23 (1.16-1.58)** | **1.15 (1.08-1.23)** |
| 24+ | **1.40 (1.24-1.58)** | **1.22 (1.08-1.39)** |
| **ASA score** | | |
| **1-2** |  |  |
| 2-4 | **0.77 (0.70-0.84)** | **0.75 (0.68-0.83)** |
| 5-8 | **0.87 (0.81-0.93)** | **0.86 (0.80-0.92)** |
| 9-12 | Ref | Ref |
| 13-23 | **1.11 (1.04-1.19)** | **1.08 (1.01-1.16)** |
| 24+ | **1.38 (1.21-1.57)** | **1.31 (1.15-1.50)** |
| **3-5** |  |  |
| 2-4 | **0.78 (0.73-0.84)** | **0.87 (0.81-0.94)** |
| 5-8 | **0.84 (0.80-0.88)** | **0.88 (0.84-0.93)** |
| 9-12 | Ref | Ref |
| 13-23 | **1.14 (1.09-1.20)** | **1.09 (1.04-1.14)** |
| 24+ | **1.26 (1.16-1.37)** | **1.16 (1.06-1.25)** |

Model 1: adjusted for age and sex, Model 2: additionally adjusted for walking ability before the fracture, ASA score, CCI, complications during hospitalization, and living arrangements.
